# Emerging hemostatic materials for non-compressible hemorrhage control

**DOI:** 10.1093/nsr/nwac162

**Published:** 2022-08-17

**Authors:** Ruonan Dong, Hualei Zhang, Baolin Guo

**Affiliations:** State Key Laboratory for Mechanical Behavior of Materials, and Frontier Institute of Science and Technology, Xi’an Jiaotong University, Xi’an 710049, China; State Key Laboratory for Mechanical Behavior of Materials, and Frontier Institute of Science and Technology, Xi’an Jiaotong University, Xi’an 710049, China; State Key Laboratory for Mechanical Behavior of Materials, and Frontier Institute of Science and Technology, Xi’an Jiaotong University, Xi’an 710049, China; Key Laboratory of Shaanxi Province for Craniofacial Precision Medicine Research, College of Stomatology, Xi’an Jiaotong University, Xi’an 710049, China

**Keywords:** non-compressible hemorrhage control, commercialized hemorrhage-control strategies, hemostatic sponge, hydrogel, microparticle, platelet mimics

## Abstract

Non-compressible hemorrhage control is a big challenge in both civilian life and the battlefield, causing a majority of deaths among all traumatic injury mortalities. Unexpected non-compressible bleeding not only happens in pre-hospital situations but also leads to a high risk of death during surgical processes throughout in-hospital treatment. Hemostatic materials for pre-hospital treatment or surgical procedures for non-compressible hemorrhage control have drawn more and more attention in recent years and several commercialized products have been developed. However, these products have all shown non-negligible limitations and researchers are focusing on developing more effective hemostatic materials for non-compressible hemorrhage control. Different hemostatic strategies (physical, chemical and biological) have been proposed and different forms (sponges/foams, sealants/adhesives, microparticles/powders and platelet mimics) of hemostatic materials have been developed based on these strategies. A summary of the requirements, state-of-the-art studies and commercial products of non-compressible hemorrhage-control materials is provided in this review with particular attention on the advantages and limitations of their emerging forms, to give a clear understanding of the progress that has been made in this area and the promising directions for future generations.

## Teaser

This review summarized the requirements, commercialized strategies and state-of-the-art studies of hemostatic materials for non-compressible hemorrhage control, providing a progress and future directions of this area.

## INTRODUCTION

Hemorrhage control is a long-term problem that has been studied from generation to generation in human history [1–3] and one of the big challenges in hemorrhage control is non-compressible bleeding [4,5]. Each year, traumatic bleeding causes ∼1.5 million deaths globally [6] and non-compressible bleeding is responsible for 90% of military traumatic injury deaths and accounts for 30%–40% of those happening in civilian life [7]. It is important to develop a hemostatic material that can effectively achieve rapid bleeding control in a non-compressible hemorrhage, which will save many lives in both civilian life and on the battlefield.

**Figure 1.**
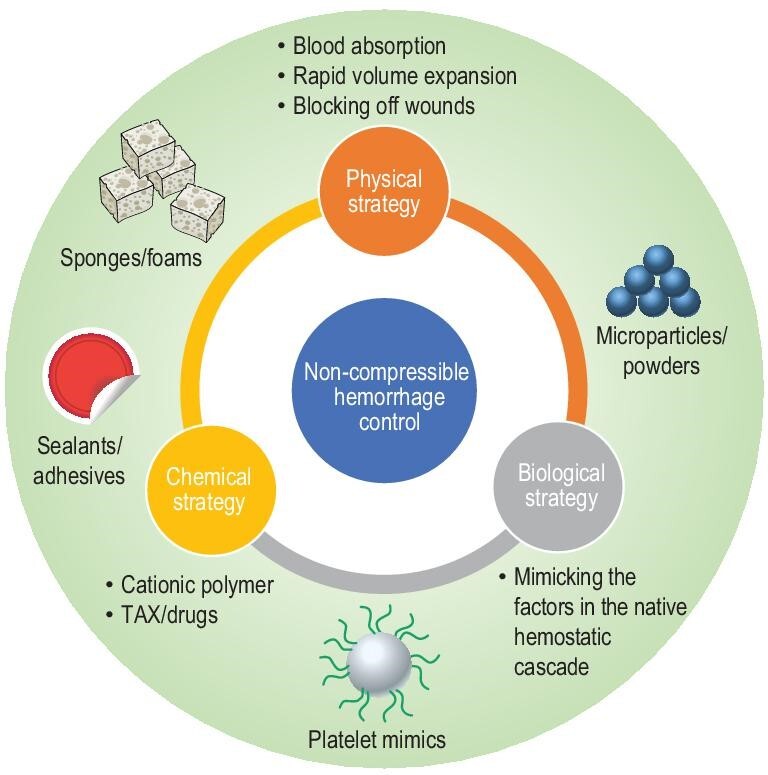
Strategies and different forms of materials for non-compressible hemorrhage control.

In the past decade, the development of hemostatic materials for non-compressible hemorrhage control has met its growth stage. Various hemostatic materials have been developed [2,3,8–18] and some of them have already been commercialized [7,19–21]. Different kinds of hemostatic materials have been designed and they work via different hemostatic mechanisms, including physical, chemical and biological approaches (Table 1). The physical strategy is through the expansion of the hemostatic reagent to generate compression on the bleeding site, reducing the blood flow to achieve hemorrhage control [9,22]. Blocking off the bleeding site by sealing the wound is another physical way [3,18,23,24]. Concentration of the blood cells and coagulation factors through physical absorption of the blood also works in stopping bleeding [15,25–27]. Chemicals with specific structures or properties, such as cationic polymers, can trigger the native hemostatic process and accelerate hemostasis as well [28–31]. In biological terms, materials that mimic the structure and/or the function of hemostatic factors and work on the native hemostatic process, such as platelets, have been developed to control bleeding [32–35]. Based on the different hemorrhage-control mechanisms, different kinds of materials have been developed, including sponges/foams [36–44], sealants/adhesives [3,14,17,18,24,45–50], microparticles/powders [15,27,51–54] and platelet mimics [32–35,55–59] (Fig. 1). Each kind of hemostatic material has its advantages and limitations and a hemostatic material that can meet all the needs for non-compressible hemorrhage control is still lacking.

**Table 1.** Hemostatic strategies and delivery methods of different forms of hemostatic materials for non-compressible hemorrhage control.

Forms of hemostatic materials	Hemostatic strategies	Delivery methods
Sponges/foams	1. Physical strategy (compression)2. Chemical strategy (blood concentration, activate coagulation cascade)	1. Direct application to the wound site by packing2. Injection with a specific syringe to the penetrating wounds
Sealants/adhesives	1. Physical strategy (seal the bleeding site, block bleeding)2. Chemical strategy (active ingredient that can trigger the native hemostatic process)	1. Direct application to the wound site by adhesion2. *In situ* formation on the wound site by injection (liquid status) and gelation via a specific method (directly gelation, photo-cross-linking, etc.)
Microparticles/powders	1. Chemical strategy (active ingredient that can trigger the native hemostatic process)2. Physical strategy (absorb blood)	1. Spraying with specific devices2. Directly apply to the wounds3. Self-driven movement to the bleeding site by force of gas generation
Platelet mimics	Biological strategy	Vein injection

Understanding the requirements for non-compressible hemorrhage-control materials and the progress and challenges in their development is important for the design and fabrication of the next generation of hemostatic materials for non-compressible hemorrhage control. Therefore, in this review, we give a systematic summary of the requirements and design principles of non-compressible hemorrhage-control materials, the current remarkable work and progress that have been done in this area and the advantages and limitations of each kind of hemostatic material developed for non-compressible hemorrhage control. Based on the work that has been done to date, the future generation of hemostatic materials for non-compressible hemorrhage control is expected to be more effective in reducing bleeding time and volume, degradable and intelligent, fit for multiple kinds of traumatic wounds and have integrated hemostatic ability with wound-healing-promotion capability. We hope this review can give the researchers who are working on the hemostatic materials, no matter whether they are beginners or professionals, a clear picture of the current status of this field and the advantages and shortcomings of the previously developed hemostatic materials or strategies so that they do not need to reinvent the wheel.

## REQUIREMENTS AND PRINCIPLES OF HEMOSTATIC MATERIALS FOR NON-COMPRESSIBLE HEMORRHAGE CONTROL

Non-compressible hemorrhage can be divided into three categories based on the bleeding site: non-compressible torso hemorrhage (NCTH), non-compressible junctional hemorrhage and non-compressible extremity hemorrhage (NCEH) [7]. Among these kinds of non-compressible hemorrhage, NCTH is the most common and most challenging type, which is accompanied by high-grade injury in intrathoracic orintrabdominopelvic organs or vasculature [60,61]. Most deaths happen within 30 min after an NCTH due to insufficient control of the bleeding [62] since a non-compressible hemorrhage causes loss of >30% of the whole-body blood volume, leading to a systolic blood pressure of <90 mmHg [63]. Since traditional tourniquets have little success in controlling an NCTH [6], developing hemostatic materials for bleeding time and volume reduction via new hemorrhage-control strategies is of great importance. Therefore, understanding the requirements and principles of hemostatic materials for non-compressible hemorrhage control is essential.

First, the most important aim for hemostatic materials is to rapidly stop bleeding, so fast bleeding-control ability is the basic requirement. There are mainly three different ways to help accelerate bleeding control. The first one is sealing. Using sealant or adhesive that can block off the injury site and stop the bleeding is the most common strategy [50]. However, non-compressible bleeding always happens in deep wounds and inner organs, which causes two problems for the sealant to work efficiently. The first problem is the high blood pressure of the artery [45], requiring high adhesive strength of the materials. Another problem is the wet environment at the wound site, leading to a reduction in the adhesive ability of most kinds of adhesives [18,47]. Therefore, high adhesive strength in a wet environment is very challenging and is required for sealants used for non-compressible hemorrhage control. To stop or reduce bleeding, reducing the blood flow is another straightforward idea. Other than blocking off the injury site by adhesion, compressing the bleeding site using volume expansion of materials to tamp the holes also works, which is the hemorrhage-control mechanism of most sponges and foams [64]. The most challenging issue for physical compression is the speed of the expansion of the sponges [38,65]. They should also have strong mechanical strength to bear the high arterial blood pressure. Another way to effectively stop non-compressible bleeding is to use drugs or platelet-like biologicals to initiate and strengthen the innate hemostasis process [21,33]. For this strategy, the requirements for the biologicals are obvious and they need to be easy to store.

Second, other than the specific requirements for each strategy, there are still some common requirements for non-compressible hemorrhage-control materials. Because these materials are always used for first-aid and pre-hospital hemorrhage control, ease of use is the biggest request, not only for medical workers, but also for non-trained civilians. Being inexpensive is another preferred property, as well as portability.

Finally, except for the specific requirements of the materials for non-compressible hemorrhage control, there are still some basic rules in developing hemorrhage-control materials that need to be followed. Because of the *invivo* application, good biocompatibility of the materials is essential. Since hemorrhage-control agents always facilitate clot formation [66], a concern with them is that they may lead to thrombosis. In that case, a hemostatic material that can effectively trigger coagulation without inducing further thrombosis is required.

## CURRENT CLINICAL HEMOSTATIC STRATEGIES FOR NON-COMPRESSIBLE HEMORRHAGE CONTROL AND THEIR LIMITATIONS

To date, several commercialized hemostatic materials for non-compressible hemorrhage control have been applied in the clinic (Table 2). They are based on different hemostatic mechanisms and their applications are also different.

**Table 2.** Currently used commercialized hemostatic strategies for non-compressible hemorrhage control and their advantages and limitations.

Commercialized hemostatic materials and devices	Working methods	Advantages	Limitations
AAJT	Provide efficient external compression on the non-compressible abdominal aorta and junctional part	Mechanical compression stress can be controlled by inflating	This may lead to the complications of compression, such as ischemia in the intestine and liver
REBOA	Place and inflate balloon in the aorta to reduce the blood pressure at the bleeding site to stop bleeding	Fast and efficient bleeding control on the hemorrhage site	Require well-trained medical individuals to apply this technique. Damage to the vascular and the reperfusion may lead to further problems
iTClamp	Seal the wound by clamping the wound edges	Easy to apply, minimum pain	Cannot be used in the inner wounds and is hard to control deep wound bleeding
RevMedx Xstat	Rapidly expanding sponges can concentrate blood, seal the wound and block the bleeding at the injection to the wound site	Fast bleeding control for penetrating and junctional bleeding	Not degradable and needs extra work to remove from the wound site
ResQFoam	Foam that is generated once injected into the wound site and contacts the blood flow, which can compress the wound site to stop bleeding	This foam can be formed *in situ* by simple injection	The foam-forming reaction is a thermogenic process that will be harmful to the tissues. The application and removal of this foam need extra work by experienced professionals
Tranexamic acid (TXA)	Reduce blood loss and accelerate coagulation	Effective in most kinds of non-compressible bleeding when applied in time	The administration time of this drug dramatically affects its efficiency

### Abdominal aortic and junctional tourniquet (AAJT)

The basic idea for controlling NCTH is using external aortic compression; however, traditional external aortic compression usually needs a heavy weight (>200 lbs) of the performer to successfully apply compression for hemorrhage control [67]. AAJT is a kind of external compression device with a wedge-shaped bladder [68]. This device works by applying mechanical strength on the bleeding site and the pressure can be controlled by manually inflating. It has been used in the battlefield and in civilian applications as a temporary bleeding-control device and has shown good effects. However, some case reports show that this kind of device leads to the complications of compression, such as ischemia in the intestine and liver [68], and it is forbidden to be used for patients who are pregnant or have abdominal aorta aneurysms [69].

### Resuscitative endovascular balloon occlusion of the aorta (REBOA)

A technique named REBOA was developed by placing and inflating the balloon at a certain location in the aorta to block the blood flow of the aorta to stop serious bleeding. This is an efficient way of controlling non-compressible bleeding that has happened in the vascular or in the abdomen. It has been reported that using REBOA can reduce mortality as well as resuscitative thoracotomy [70,71]. A concern for this technique is that it requires trained medical persons to apply it, which are usually lacking on the battlefield and in accidents. Meanwhile, during the application of REBOA, the insertion can cause damage to the vessels, embolization, etc. and the reperfusion leads to failure of the organ [72,73].

### iTClamp

The iTClamp is a kind of easily applied clip that can seal wound edges to achieve hemorrhage control. It can seal by just clamping it to the wound and the pain caused by the iTClamp is minimal. Studies have demonstrated that the iTClamp can effectively reduce bleeding time [74,75] and improve the survival ratio [74]. However, since the iTClamp is applied externally on the wound edges, it cannot be used for inner injuries and it is hard to control deep wound bleeding.

### RevMedx Xstat

RevMedx Xstat is an FDA-approved injectable sponge that can control hemorrhage caused by penetrating wounds, such as gunshot or knife wounds. It contains sponge pellets in a syringe-like device and these sponge pellets can expand within several seconds to concentrate blood, seal the wound and block the bleeding. The Xstat is demonstrated to have remarkable performance in controlling junctional bleeding in comparison with other commercial products [76]. A recent study has shown that the Xstat 30 also works well in uterine hemorrhage control [77]. Its effect on NCTH was further studied using the swine liver lobe transection model and the results showed that the Xstat 30 can improve the survival ratio [20]. A concern is that the Xstat is not degradable and it needs to be removed from the wound site once the bleeding has been stopped. Although the developers have addressed this issue by making it traceable using embedded radiopaque markers, the complete removal of it still takes time.

### ResQFoam

ResQFoam is a kind of polymeric foam developed by Arsenal Medical. It can be formed *insitu* by injecting two kinds of liquid polymers that react with each other to generate foam and this foam can expand with the blood flow, compressing the injured site to stop bleeding. Animal tests conducted on swine low-pressure venous bleeding and high-pressure arterial injury showed that this foam could significantly enhance the survival of the animals [19]. A concern with ResQFoam is the thermo-effect of the foam-forming reaction. Also, this foam can only be injected by specifically trained personnel and removed by laparotomy, which is not very convenient for first-aid rescue.

### Tranexamic acid (TXA)

TXA is a synthetic molecule that mimics the structure of lysine and it shows anti-fibrinolytic properties [78]. Local application of TXA can help reduce blood loss and accelerate coagulation [79]. The application of TXA dramatically reduces the blood loss in off-pump coronary artery bypass surgery [80] and it also works in reducing blood loss in orthotopic liver transplantation [81]. However, the efficacy of this drug is related to the administration time, which showed fewer effects after 3 h [82].

Currently, clinically used hemostatic devices have four main limitations: poor portability, high technical requirements, insufficient in removal and effects leading to complications. Since the currently commercialized hemostatic products for non-compressible hemorrhage control all have their limitations (Table 2), better hemostatic material is desired in this field.

## EMERGING CATEGORIES AND THEIR HEMOSTATIC MECHANISMS OF HEMOSTATIC MATERIALS FOR NON-COMPRESSIBLE HEMORRHAGE CONTROL

**Figure 2.**
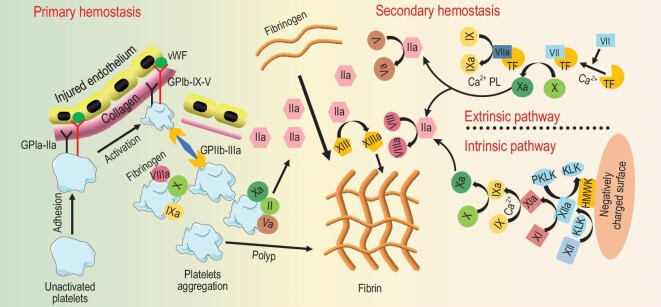
The innate hemostatic cascade of the human body and the stages of the materials function in the hemostatic cascade. The hemostatic process mainly contains two parts: the primary hemostasis and secondary hemostasis. In the primary hemostasis process, platelets are activated and aggregated. This inspired the development of hemostatic materials that trigger the intrinsic hemostatic process by promoting or mimicking platelets adhesion [32], accelerating platelet activation [94,95] and facilitating platelet aggregation [2,11,101]. The secondary hemostasis can be divided into the extrinsic pathway and the intrinsic pathway. In the extrinsic pathway, blood is exposed to tissue factors (TFs) and they finally activate factor IX to factor IXa to further help the matured clot formation. In that case, TFs and factors related to this cascade have also been involved in non-compressible hemorrhage control [96]. At the same time, the intrinsic pathway starts with factor XII and ends with the generation of factor Xa, which can form factor IIa for the fibrin network formation. The negatively charged surface can contribute to the intrinsic pathway and they have also been developed into hemostatic materials to control bleeding. Finally, factor IIa transforms factor XIII to XIIIa, which helps the fibrin to form a cross-linked fibrin network to strengthen the clotting. In some work, material that can mimic factor XIII was developed to accelerate hemostasis [97], while the platelet mimics usually work in this process to facilitate fibrin cross-linking [35].

Hemostatic materials achieve hemorrhage control via affecting the intrinsic hemostatic process of the human body. Therefore, all hemostatic strategies ultimately aim to facilitate different steps in the hemostatic process (Fig. 2). Based on different hemostatic mechanisms, different kinds of hemostatic materials that can rapidly reduce blood loss have been developed. Forms of the hemostatic materials contribute a lot to their functions [83] and the fabrication process determines the forms and structures of hemostatic materials for non-compressible hemorrhage control. By different preparation methods, hemostatic materials can be formed into high-porosity structures (such as sponges [39,84–88] and foams [39,40,51]), tough adhesive gels (such as sealants [18,24,45,48,49,89,90] and adhesives [3,16,17,46,47,50]), micro-sized structures (like microparticles [52,91] and powders [5,92,93]) and biomimetic structures (platelet-like structures [32–35,55,57,59]). However, the hemostasis strategies (physical, chemical and biological) usually are not developed specifically via a single hemostatic mechanism or target only one step of the hemostasis process. For example, physical strategies mostly involve pressing the bleeding site to reduce the blood flow and concentrating blood cells, platelets and coagulation factors. This process mainly depends on the activation of the primary hemostasis process [94,95]. But the components used in hemostatic materials can also trigger the intrinsic pathway and the extrinsic pathway to activate secondary hemostasis to promote bleeding control [96,97]. In most cases, the three different hemostatic strategies are intercalated and synergistic effects of different hemostatic mechanisms are involved. In this part, different forms of hemostatic materials for non-compressible hemostasis control are provided and their hemostatic mechanisms are discussed.

### Sponges/foams

**Figure 3.**
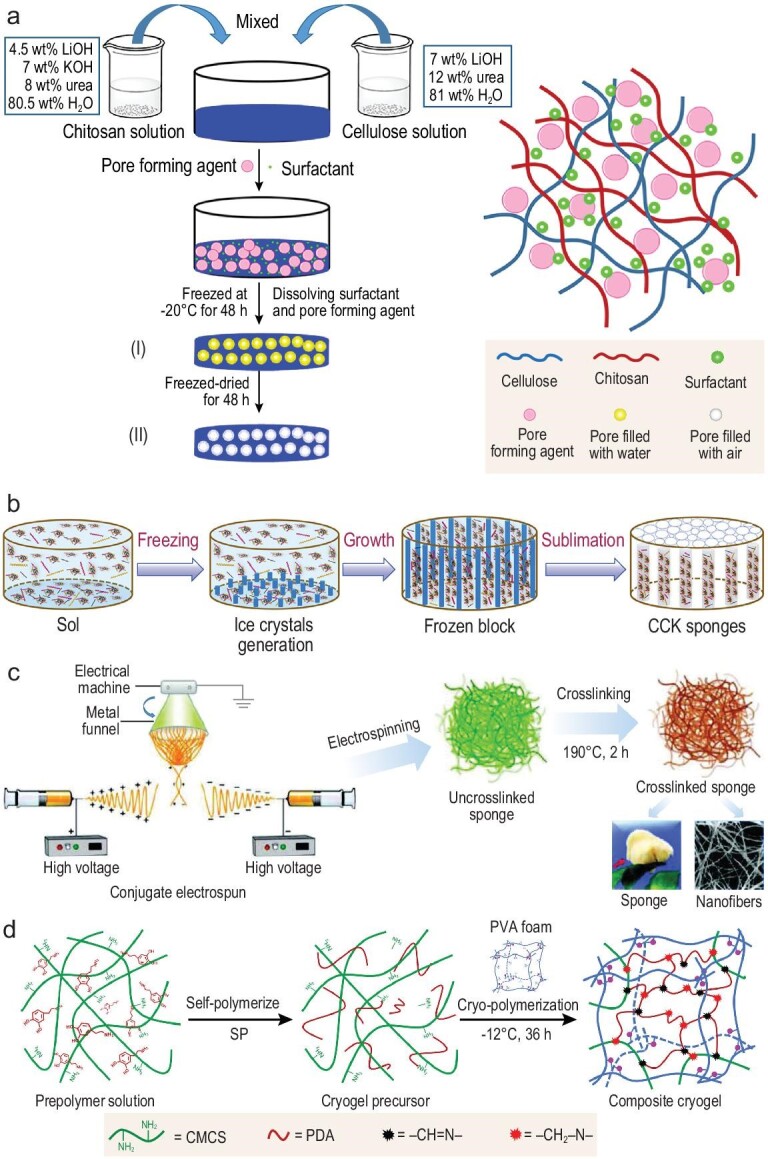
The different strategies of the formation of porous sponges. (a) Using a pore-forming agent to fabricate pores. Adapted with permission from Ref. [65], Elsevier. (b) Using the oriented freezing method to create aligned pores via the generation and sublimation of aligned ice crystals. Adapted with permission from Ref. [11], Elsevier. (c) The conjugate electrospun method can generate nanofibrous sponges by the entanglement of nanofibers with different charges and subsequent cross-linking. Adapted with permission from Ref. [37], Wiley-VCH. (d) The sponge with synergistic hemostatic effects generated via cryo-polymerization in the PVA foam. CMCS, carboxymethyl chitosan; PDA, polydopamine; SP, sodium periodate. Adapted with permission from Ref. [44], Elsevier.

A strategy for non-compressible hemorrhage control is generating compression pressure at the wound site by the materials, as well as absorbing blood cells, platelets and blood proteins to facilitate coagulation. Sponges and foams with porous structures can meet the requirements. Porous structures in sponges and foams enable them to have high absorbing capacity for blood and also make them low in density and light in weight. Most sponges with porous structures can be compressed into a small size and can expand when absorbing liquids. A high surface area and porosity are of great importance for hemostasis and the porous structure enhances the surface area of the sponges and foams efficiently. To obtain the porous structure, researchers have developed several different approaches. The easiest one is using a pore-forming agent, such as Na_2_SO_4_ (Fig. 3a) [65]. The most commonly used method is lyophilization [8,9,98,99]. Self-foaming is another easy way. A sponge is formed by self-foaming of hyaluronic acid (HA) and poly((2-dimethyl amino)-ethyl methacrylate)-grafted dextran (Dex-PDM) or partially-quaternized Dex-PDM with sodium trimetaphosphate as a crosslinker via mixing using an egg beater [100]. The self-foaming is claimed to be caused by the viscous/amphiphilic HA/Dex-PDM complex, as a foaming agent, generated via the electrostatic effects of the HA and Dex-PDM. The 3D printing method is another way to generate sponges with unified porous structures and 3D printed sponges also show rapid shape-recovery properties as well as blood-absorption behavior [101].

By directly freeze-drying cross-linked hydrogels, sponges or cryogels with evenly distributed pores can be generated, caused by the phase separation, leaving voids by the sublimation of the ice crystals in the hydrogels [102]. Furthermore, by orientation freezing, the pore can be produced in an oriented and interconnected way (Fig. 3b), which can obtain a capillary-structured sponge and improve the blood-absorbing capability to further promote the coagulation of the plasma [11]. The porosity of the sponges formed by lyophilization methods can range from ∼80% to ∼97%, achieving a low weight and high surface area. Another approach that is worth mentioning is the one proposed by Xie *et al.* [37], which can generate a high-porosity sponge with nanofibrous structures. In their work, positively charged and negatively charged gelatin nanofibers were electrospun together to generate nanofibrous sponges by the entanglement of the differently charged nanofibers (Fig. 3c). This 3D sponge showed high compressibility and water-absorption capacity. Meanwhile, the gelatin nanofibers in this sponge can aggregate and activate platelets to accelerate the instinct hemostatic process of the body. Chen and colleagues incorporated carboxymethyl cellulose (CMC) onto a hydrogen-bonding *N*-acryloyl-2-glycine (ACG) monomer to facilitate a heat-initiated polymerization and obtained a robust sponge [8]. The porous structure is achieved by vortexing the monomer solutions to introduce air bubbles and subsequent lyophilization. Vortexing to introduce bubbles into the solution is a simple way to generate porous structures, although the sizes of the pores are hard to control and the viscosity of the polymer solution affects the generation of the bubbles greatly, which needs to be controlled carefully.

Except for the blood-absorption effect, sponges can also work for hemorrhage control through their shape-adaptive effects to expand and compress the bleeding site. For this property, high compressibility and shape-adaptive capacity are required from the sponges. Using shape-memory polymers can achieve this goal. Beaman *et al.* developed a shape-memory polymer foam that can be stored in a compressed state by heating the pre-formed sponge to a temperature higher than its glass transition temperature, compressing and cooling [39]. When the compressed sponge is exposed to blood at body temperature, it will recover to its original shape, which is an expanded state, and fill the injury space to compress the wound site, achieving hemorrhage control. This sponge shows similar blood-loss time to Xstat and QuikClot^®^ Combat Gauze, but the bleeding time is reduced when treated using this sponge. This sponge treatment also shows significantly improved survival of swine with liver injury. However, in this study, the authors used the powders of the sponges to do the *in vivo* hemostatic testing instead of directly using the shape-memory sponge. The effect of the material on hemostasis in a sponge form still needs to be determined.

Cryogels also exhibit a fast compressed-to-expanded state change. Our group has developed a series of cryogel sponges as hemostatic agents for non-compressible hemorrhage control [42,43,103]. Using glycidyl methacrylate functionalized quaternized chitosan as the main component together with carbon nanotubes, the cryogel was formed by freezing the precursor solution and lyophilization subsequently [42]. This cryogel exhibited robust compression properties as well as rapid blood-absorption behavior, due to its interconnected porous structure. Meanwhile, quaternized chitosan endowed this sponge with antibacterial properties and the photothermal property of the carbon nanotubes (CNTs) also contributed to infection-control behavior when irradiated with near infrared (NIR). Importantly, this conductive hemostatic sponge showed remarkable blood concentration and high blood cell and platelet aggregation and activation behavior, which also led to fast bleeding-control behavior in the rabbit liver defect lethal non-compressible hemorrhage model. However, the CNTs’ role in the blood cell and platelet activation of this sponge is worthy of further study and how the porosity affects the hemostatic behavior of the sponges should be addressed in future work, which is important for the development of hemostatic sponges. Further study on the effects of this kind of cryogel sponge on lethal non-compressible hemorrhage control in big animal models was conducted using a dry cryogel based on chitosan and polydopamine [43]. The dry cryogel can effectively stop the bleeding of the swine subclavian artery and vein complete transection model, which endures rapid blood loss of ∼750 mL within 30 s. By applying this cryogel, the bleeding can be completely stopped within 4 min, among which 40% was stopped immediately after the cryogel treatment. This is a remarkable result since it demonstrated that this sponge can bear the high blood pressure of arterial bleeding and rapidly control the hemorrhage, which is practical in pre-hospital application on the battlefield and with accidental trauma. This hemorrhage-control sponge is also degradable *in vivo*, which avoids the taking-out concern. A similar porous sponge based on gelatin was developed with better degradability and biocompatibility [12]. Additionally, a combination method of foaming and cryo-polymerization was used to generate a sponge with both chemical and physical hemostatic properties to achieve rapid hemorrhage control [44]. Polyvinyl alcohol (PVA) foam was soaked in the cryogel precursor solution of carboxymethyl chitosan (CMCS) and dopamine (DA) and the cryo-polymerization was conducted under frozen conditions to generate the cryogel network within the PVA foam (Fig. 3d). PVA foam works well in absorbing blood upon application and the CMCS network can facilitate the activation of platelets in a chemical way, which synthetically can speed up the bleeding control. The combination of different hemostatic strategies is an advanced idea to generate hemostatic materials for non-compressible hemorrhage control.

Hemostatic sponges for non-compressible hemorrhage control have been well studied and the advantages of using hemostatic sponges are that they can be applied directly to the wound site, even inner ones, and can facilitate bleeding control in a synthetic manner of mechanical and chemical approaches. However, sponges absorbing blood and the coagulation of the blood inside the sponge would lead to the thickening of the sponge, strengthening its compression on the surrounding tissue and nerves, which could lead to complications. Also, the taking-out of the sponges is another concern.

### Sealants/adhesives

**Figure 4.**
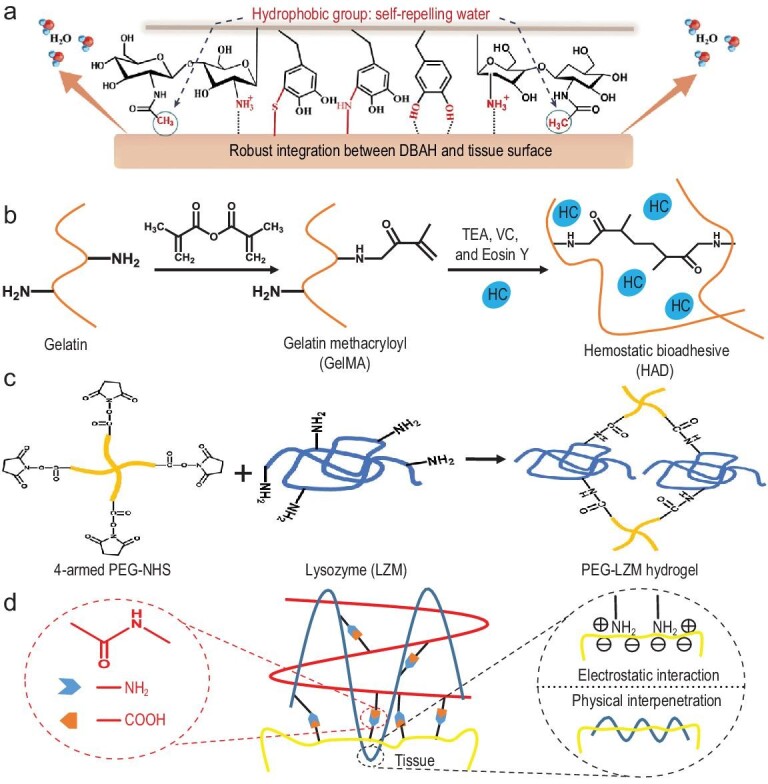
Fabrication strategies for sealants and adhesives. (a) Tissue adhesive fabricated via mussel-inspired reaction and water-repelling function of the hydrophobic groups. Schiff-base and Michael addition reactions between the catechol groups in the hydrogel and amino groups and thiol groups on the tissue surface enable the adhesiveness of the hydrogel on the tissue. The hydrophobic part of the polymers repels water from the contact surface, strengthening the adhesion. Adapted with permission from Ref. [50], Elsevier. (b) Hemocoagulase (HC)-embedded adhesive hydrogel is prepared by photo-cross-linking using Eosin Y as a photosensitizer that enables the gelation of the sealant upon visible light exposure. Adapted with permission from Ref. [46], AAAS. (c) Four-armed PEG modified with NHS reacts with lysozyme via the reaction between NHS and NH_2_ to form a hydrogel and the NHS also can react with the amino groups in the tissue to achieve strong adhesiveness on the tissues. (d) The bio-inspired adhesive relies on the electrostatic interaction, physical interpenetration, chain entanglement and covalent bond formation of the ‘bridge polymer’ with the tissues.

As mentioned above, sealants and adhesives are another kind of effective hemorrhage-control agent that has been widely studied to date [104–107]. A widely accepted problem of sealants for non-compressible hemorrhage control is that they show poor adhesive strength on wet surfaces and weak mechanical properties. To solve this problem, researchers have attempted several different kinds of approaches to obtain tough adhesives for wet surfaces. The most widely used one is the mussel-inspired strategy, using catechol side branches to achieve the tissue-adhesive goal [17,18,24,47,48,50,105]. A lot of small molecules possess catechol groups, such as dopamine and tannic acid, and they can react with amino groups and thiol groups on the surface of the tissues through a Schiff-base reaction and Michael addition reaction. Studies have demonstrated that liquid coacervation is important in achieving underwater adhesion and using hydrophobic molecules modified with catechol groups can repel water on the adherent surface because of the hydrophobicity of the molecules (Fig. 4a) [50] and lead to strong adhesion due to the good exposure of the catechol groups [17]. The strongest adhesion strength to tissues (to the bone in this study) underwater of the water-triggered adhesive is 270 kPa when kept underwater for 12 h [17]. Water-triggered or water-repelling mussel-inspired adhesives can be applied directly to the bleeding site and the *in vivo* testing of their applications on the transection liver and bleeding heart showed their great potential in the hemorrhage control of orintrabdominopelvic organs. Except for the mussel-inspired adhesion, photo-cross-linking adhesives also show noticeable performance. By modifying biomacromolecules with glycidyl methacrylate (GMA), the biomacromolecules can be endowed with photo-cross-linking properties [45]. Upon applying to the bleeding site, the pre-cross-linking agent was a solution, although, when illuminated with light of a proper wavelength, the agent cross-links to a gel form and seals the wound together. Since the agent is in a liquid state before application, it can be used for sealing injuries to inner organs via an endoscopic device. Besides, by incorporating components that can facilitate or accelerate the native hemostatic process, such as accelerating platelet aggregation, in the sealants, they can effectively control non-compressible bleeding [46]. For example, it was demonstrated that snake extract can induce rapid blood-stopping, activate platelets and accelerate fibrin formation [108]. By lading hemocoagulase into a photo-cross-linkable hydrogel, a sealant that can rapidly facilitate clotting, blood-cell aggregation and platelet activation was fabricated [46]. This photo-cross-linking hydrogel used Eosin Y as the photosensitizer and this sealant can be cross-linked via visible light, reducing damage to the tissues caused by UV exposure (Fig. 4b). Integration of a photo-cross-linking strategy with the mussel-inspired adhesive strategy gains a foothold in the non-compressible hemorrhage-control area, and possesses rapid gelling behavior and strong adhesiveness to the tissues [3]. On applying it to a bleeding heart and damaged arteries, the hydrogel sealant can bear a blood pressure of ≤290 mmHg. Application of this sealant in swine heart penetrated with an incision of 6 mm can stop bleeding quickly and dramatically enhance survival. This sealant is promising in clinics for traumatic wound-induced non-compressible bleeding control, although *in vivo* safety still needs to be further studied and the photosensitizer can be changed to avoid the use of UV light.

The formation of amide links can also generate hemorrhage-control sealants with strong wet-tissue-adhesive ability. Multi-armed polyethylene glycol (PEG) is desirable in the sealant formation since it shows good water solubility, no immunogenicity and bio-absorbability. *N*-hydroxy succinimide-modified four-armed PEG (four-armed PEG-NHS) was used to fabricate an adhesive hydrogel with the interaction of the residual succinimidyl-active ester with the amide groups on the tissue [90]. However, PEG showed non-cell-adhesive properties that are not compatible with cell attachment and tissue regeneration. Therefore, the adoption of biomacromolecules that possess an anchorage site for cell attachment is desirable. By adding lysozyme to the four-armed PEG-NHS, they can cross-link into a hydrogel with a reaction between the NHS and the NH_2_ groups in the lysozyme (Fig. 4c) [49]. This sealant showed similar adhesive properties to the four-armed PEG-NHS hydrogel, although it has better cell affinity and antibacterial properties that are beneficial for the wound-healing process subsequently to the hemorrhage control.

To obtain strong adhesive ability, hydrogels with multiple adhesive strategies play a conspicuous role. An adhesive that possesses covalent bonds, physical interpenetration, electrostatic interactions, as well as amplified energy dissipation was developed to generate strong adhesion to native tissues [16]. A polymer with multiple NH_2_ groups was chosen as a so-called ‘bridging polymer’ that can not only form covalent bonds with the carboxylic acid groups on the tissue surface, but also penetrate the tissue to achieve physical entanglement via electrostatic attraction after being protonated (Fig. 4d). The dissipation layer of this sealant enables it to have a toughened interface, leading to an increase in the adhesive energy, which can be ≤1 kJ/m^2^, on the skin. However, the safety issue of *in vivo* application remains since a multiple coupling reagent was involved.

A problem for adhesives/sealants is that strong adherent strength and easy removal are always mutually exclusive. The removal of a strongly adherent sealant, especially after the coagulation of the blood happens at the wound site, frequently leads to pain and secondary bleeding. To solve this problem, an adhesive with a triggerable benign detachment was developed [109]. This adhesive adheres to the tissue surface via two time-dependent effects: at the beginning, after application, PVA in the dry adhesive will absorb the water on the surface and the instant adhesion is achieved by the physical interaction (such as the hydrogen bond) formed between the carboxyl acid group in the poly(acrylic acid) (PAA) in the adhesive and the tissue surface. As time goes by, the NHS, which is linked to the PAA with a disulfide bond, generates a covalent cross link with the amino groups on the tissue surface. The adhesive can be triggered to detach from the tissue surface by molecules that work on breaking the physical and covalent cross-linking, respectively. Sodium bicarbonate is used to change the pH, disrupting the hydrogen bond, and biocompatible reducing agents can be used to break the disulfide bond, enabling the benign detachment of the adhesive. The on-demand removal property is desirable but the detachment-triggering agent should be carefully chosen to make sure that it will not affect the formed clot and the subsequent treatment of the wounds. Other researchers have changed their minds by replacing the hemostatic materials with a wound-healing dressing to endow the sealants/adhesives with wound-healing-promotion properties. Thus, the sealant plays a versatile role as both a hemostatic agent and a wound-healing dressing.

A limitation for adhesives is that although they can effectively seal superficial wounds and exposed wounds (such as surgical wounds), they are not suitable for penetrating wounds that are always deep and sharp (such as hemorrhage caused by a gunshot). Even for exposed wounds, in most studies conducted to date, the efficiency of sealants/adhesives on aorta hemorrhages with high blood pressure and a large volume of bleeding has not been well investigated. Although more and more adhesives with high adhesive strength to wet surfaces have been developed, tests of their adhesiveness were mostly conducted on a static wet surface or on a bleeding model with low blood pressure. Their effects *in vivo* on aorta hemorrhage models of big animals (such as swine or goat) are still lacking.

### Microparticles/powders

**Figure 5.**
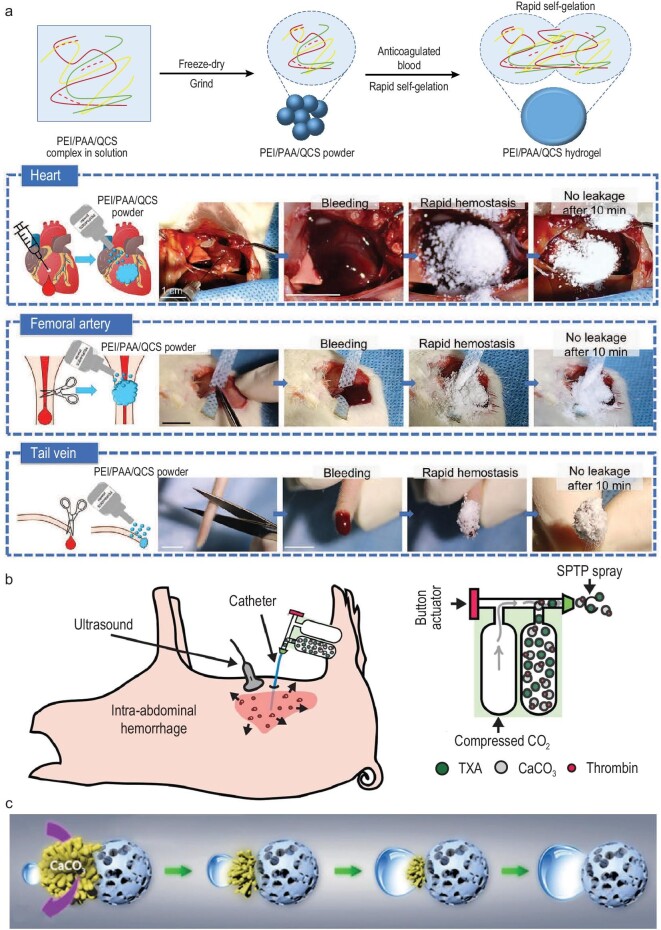
Examples of rapid-gelation hemostatic powders and a device for the application of intra-abdominal hemorrhage-control microspheres. (a) The PEI/PAA/QCS powder formed via the freeze-drying and grinding of the PEI/PAA/QCS complex. This powder can absorb blood at a very fast speed with self-gelation into hydrogels subsequently to stop bleeding. It can be applied as the powder form to the wound site at the heart, femoral artery and the tail vein directly and achieve fast bleeding control. Adapted with permission from Ref. [5], Wiley-VCH. (b) The device that enabled the intra-abdominal application of the hemostatic powders via the compression of CO_2_. Adapted with permission from Ref. [93], Elsevier. (c) Bubbles generated resulting from the dissolving of the CaCO_3_ and TXA in the Janus microparticles and the detachment action of the bubbles from the microparticles will drive the motion of them. Adapted with permission from Ref. [110], Wiley-VCH.

For irregularly shaped wounds, microparticles or powders are desirable as hemostatic agents since they can be applied directly to wounds to fill irregular shapes and absorb blood to concentrate coagulation factors, achieving effective hemorrhage control. The key factor for microparticles/powders for hemorrhage control is their blood-absorption behavior. Surface morphology can contribute greatly to their blood-absorption capacity. By emulsion and freeze-drying, microspheres with lotus-seedpod-like structures can be generated. There are micropores in the micropits on the microspheres, which lead to the rapid blood-absorption behavior of microspheres [15]. Besides the surface structures, a high swelling ratio is another desirable property of microspheres. A polyethyleneimine/polyacrylic acid/quaternized chitosan (PEI/PAA/QCS) powder was developed with rapid gelling and wet-adhesive behavior. PAA has different charges to PEI and QCS, and when their solution is mixed together, the polymer chains will entangle via electrostatic interactions to form a PEI/PAA/QCS complex. By freeze-drying and grinding the complex, the PEI/PAA/QCS powder will be generated (Fig. 5a). This powder can absorb blood quickly and form a hydrogel within 4 s [5]. The effective absorption of blood achieved the concentration of coagulation factors and the formed hydrogel showed strong adhesive strength (about twice normal blood pressure) to tissues such as the liver, heart and spleen wetted with anticoagulated blood. Applying this powder to non-compressible bleeding sites, including the heart, femoral artery and tail vein, can achieve hemorrhage control within 10 s (Fig. 5a). The PEI/PAA/QCS powder can not only form the hydrogel adhesive at the bleeding site as a barrier, but also can concentrate coagulate factors and aggregate blood cells and platelets, leading to the acceleration of hemostasis. This powder can be applied to wounds that can be seen directly but for hemorrhages that have happened in inner organs such as a non-compressible intra-abdominal hemorrhage, the application of powders needs a new strategy. A self-propelling thrombin-containing powder was developed that can be applied intra-abdominally via a catheter-spray applicator [93]. CaCO_3_ microparticles formulated with thrombin and TXA were stocked in the spray bottle and CO_2_ was contained in the other bottle connected to the microparticle bottle using a catheter (Fig. 5b). When compressing the CO_2_ bottle, the airflow enabled the microparticles to be sprayed onto the wound site evenly, covering a wide area. This device enables the inner application of the microparticles and powders. Apart from using devices to achieve widespread application of the particles, self-propelling Janus hemostatic microparticles are designed for device-free spreading of hemostatic agents [110]. A negatively modified microporous starch was established and modified using CaCO_3_ crystals on it by uniaxial growth. Thrombin was loaded onto this particle and TXA-NH_3_^+^ was mixed with CaCO_3_ at the same molecular ratio. Upon contact with the liquid, the TXA and CaCO_3_ will be dissolved quickly, forming bubbles. The self-propelling motion is generated by the detachment of the bubbles that were growing on the Janus particles (Fig. 5c), driving the particle to move to the deep part of the wound, facilitating coagulation at that site with the thrombin and drugs loaded into the particles. Advantages of microparticles/powders for non-compressible hemostatic control are that they can fit irregular wounds, get deep into the wound site and cover a large wound area with even distribution. But the concern is their removability: if they go deep into the wound and vascular, will they be in the circulation system and stack at unexpected sites, such as capillaries, and cause complications? Therefore, the biggest challenge in the application of microparticles/powders is solving the hard-to-completely-remove issue.

### Platelet mimics

**Figure 6.**
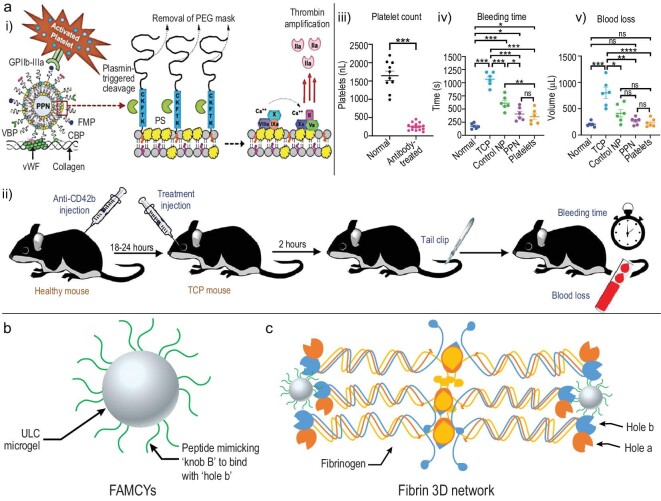
Structures of platelet mimics and how they work in facilitating hemostasis. (a) (i) Liposome modified with clearable peptide-linked PEG mask. Once this platelet mimics exposure to the plasmin, the peptide will be broken, removing the PEG mask and exposing the phospholipid phosphatidylserine (PS) on the liposome (yellow part in the scheme) which will trigger the downstream hemostasis cascade and amplification of the thrombin. VBP: a peptide that works in adhesion to von Willebrand Factor; CBP: a peptide that works in adhesion to collagen; FMP: a peptide that works in fibrinogen (Fg) binding to integrin α_IIb_β_3_ (glycoprotein GPIIb–IIIa). (ii–v) These platelet mimics can effectively reduce bleeding time and blood loss as well as the platelets did when applied by injection before the injury happened. Adapted with permission from Ref. [33], AAAS. (b) Platelet-like microgels that can facilitate 3D fibrin network formation. The ultrasoft microgels are modified using peptides that mimic the ‘knob B’ structure of the activated fibrinogen, named as FAMCYs. Adapted with permission from Ref. [35], Wiley-VCH. (c) The 3D fibrin network that formed via the binding of FAMCYs with the ‘hole b’ structures of the fibrinogen. Adapted with permission from Ref. [35], Wiley-VCH.

In the clinic, an effective method to control bleeding is the transfusion of platelets. However, platelets are bioproducts that have a short shelf-life, high requirements for storage and transportation, and are easily contaminated, limiting their application in civilian accidents and battlefields. Researchers are trying to produce platelet mimics to overcome the drawbacks of the native platelets without compromising their functionalities. Using modified liposomes as platelet mimics is a promising method. Sen Gupta's group has developed a series of synthetic platelet mimics and demonstrated their high efficacy in non-compressible hemorrhage control [32,33,57,59]. By modifying the surface of liposomes with collagen-binding, VWF-binding and fibrinogen-mimetic peptides, these liposomes showed adhesion and aggregation behavior similar to the native platelets, with high efficiency in hemorrhage control in a mouse tail transection bleeding model of normal mice [57] and thrombocytopenic mice [55], as well as in the pig femoral artery injury [32]. Furthermore, they developed platelet-mimicking procoagulant nanoparticles (PPNs) that can expose phospholipid phosphatidylserine when triggered with plasmin, subsequently facilitating the aggregation of coagulation factors on the surface of PPNs, strengthening the thrombin generation and fibrin formation (Fig. 6a i) [33]. PPNs can significantly reduce blood loss in a traumatic hemorrhage model (Fig. 6a ii–v). Liposome-based platelet mimics are easy to obtain and the modification of liposomes is effective, although the stability of liposomes is a concern. Instead of using liposomes as the framework, using microgels can achieve stable and low-cost platelet-like agents for hemorrhage control. Brown and colleagues, using poly(*N*-isopropylacrylamide-co-acrylic acid), fabricated a series of deformable microgels to act as platelet mimics to facilitate rapid hemorrhage control. The first generation of these platelet-like microgels was modified using fibrin antibodies, which can specifically bind to fibrin and facilitate the retraction of the clot [34]. However, the monoclonal antibody or their single domain variable fragment chain (sdFv) can only be generated by recombinant production, which has a high cost and low repeatability. In that case, further work using peptides replacing the monoclonal antibody or sdFv was established [35]. They generated the same microgel framework with the decoration of peptides that can mimic the structure of the fibrin knob ‘B’ (Fig. 6b) and target the fibrin hole ‘B’ to facilitate fibrin binding to the microgels (Fig. 6c). This microgel can increase the density of clots and reduce the bleeding time *in vivo*. Their application in non-controllable hemorrhage *in vivo* is needed for further study.

No matter whether using liposomes or microgels as the framework, the key factor for fabricating platelet mimics is the peptides modified on them. Several categories of peptides have been employed and demonstrated to be effective in the studies to date, such as the collagen-binding peptide, the VWF-binding peptide, the fibrinogen-mimetic peptide and the fibrin-targeting peptide. With better understanding of the mechanism of how platelets induce the instinct hemostatic process, more effective platelet-like hemostatic agents will be developed. Therefore, study of the role of platelets and their reaction with upstream and downstream factors in the hemostatic cascade is important for the development of platelet mimic hemostatic agents.

Different forms of hemostatic materials for non-compressible hemorrhage control have been developed and they have specific applicable qualities but share similar hemorrhage-control strategies. For example, sponges and foams can expand quickly and concentrate the blood due to their high porosity, which makes them suitable for penetrating wound bleeding, while microparticles and powders are suitable for irregularly shaped wound bleeding control based on their small size and good blood-absorption capacity. The chemical strategy is not too relevant with the forms of the materials. For instance, chitosan, a cationic polymer that has been reported to be effective in hemorrhage control, has been used in developing different forms of hemostatic materials, including sponges, adhesives and microparticles. And the chemical strategies together with the form-provided physical strategy synergistically achieved the hemorrhage control of non-compressible bleeding.

## CONCLUSION AND OUTLOOK

Non-compressible hemorrhage control is a tough challenge in civilian life and on the battlefield, leading to the major portion of deaths in traumatic injuries. To date, several different kinds of hemostatic products have been available for non-compressible hemorrhage control, but they all have their limitations. Therefore, the development of materials for non-compressible hemorrhage control still draws much attention from researchers. The requirements for hemostatic materials for non-compressible bleeding are strict. First of all, they need to be effective in reducing the bleeding time and volume. Meanwhile, they should not trigger complications such as downstream organ dysfunction caused by ischemia. A good hemostatic material should also fit different kinds of bleeding sites, including penetrated wounds, vascular damage, irregularly shaped wounds and combinations of these wounds. Additionally, since the hemostatic materials for non-compressible hemorrhage control are always used in pre-hospital treatments, they should be easy to carry and apply by non-trained persons. Biocompatibility, easy removal or degradability and affordability are also important for hemostatic materials for non-compressible hemorrhage control.

Based on the requirements, several new kinds of hemostatic materials have been developed, including sponges/foams, sealants/adhesives, microparticles/powders and platelet mimics. They achieve the hemostatic goal via different strategies and there are outstanding ones in each category. However, a challenge is that they still cannot meet all kinds of bleeding. A combination of different kinds of hemorrhage strategies can be a solution and physical compression together with chemical and biological triggering of the intrinsic hemostatic process can improve the efficiency of hemorrhage control, which can be a future direction. Since the development of hemorrhage control relies on both progress in biomaterials design and the study of biological mechanisms, the understanding of how the intrinsic hemostatic process happens is important and the development of more mimics of the factors in the hemostatic process can be a promising direction for the design of non-compressible hemorrhage-control materials.

Removal of the hemostatic materials applied for non-compressible hemorrhage is another challenge since they always unite as one with the clot once the bleeding has stopped (sponges and adhesives) or enter the circulation system unexpectedly (such as microparticles and powders). Therefore, hemostatic materials with on-demand and benign removal behavior are desirable. Alternatively, complete degradability within a proper time can be a focus in the future design of hemostatic materials.

Hemostasis is the beginning process of wound healing and if the materials used for hemostasis can also contribute to wound-healing promotion, it would be a once-and-for-all treatment. Some of the chemicals that can promote hemostasis were proved to be effective in promoting wound healing as well, such as chitosan. Future work can focus on using these kinds of chemicals to develop hemostatic materials with wound-healing abilities.

Hemostatic materials that can monitor bleeding conditions have also drawn much attention since they can report the status of the patients in a timely way and play a role in intelligent medical development. The design of a hemostat with such properties will probably need to combine chemistry with material science and with electronics, which will be a new direction for the development of a novel smart hemostat.

Besides the design and formation of hemostatic materials for non-compressible hemorrhage control, the evaluation methodology for hemostatic effects also needs to be standardized. Standard *in vivo* testing is very important to evaluate and compare the efficiency and hemostatic ability of hemostatic materials. However, development in this area is tentative and still lacks a standard model for testing. Most studies now use different kinds of animal models to evaluate hemostatic effects, making it hard to compare. It is also a challenge for the development of hemostatic materials for non-compressible hemorrhage control.

In a summary, although hemostatic materials have been developed for a long time, a hemostatic material that can meet all the requirements in non-compressible hemorrhage control is still lacking. Future studies should pay more attention to the development of hybrid hemorrhage-control materials to meet different requirements of non-compressible hemorrhage-control materials and deeper studies on the biological process of intrinsic hemorrhage control are needed.
